# Drug fever induced by antibiotics of β-lactams in a patient after posterior cervical spine surgery—A case report and literature review

**DOI:** 10.3389/fsurg.2022.1065106

**Published:** 2023-01-11

**Authors:** Yunxiang Hu, Jun Han, Lin Gao, Sanmao Liu, Hong Wang

**Affiliations:** ^1^ Department of Orthopedics, Dalian Municipal Central Hospital Affiliated of Dalian Medical University, Dalian, China; ^2^ School of Graduates, Dalian Medical University. Dalian, China; ^3^ Department of Spine Surgery, The People's Hospital of Liuyang City, Changsha, China

**Keywords:** drug fever, infectious disease, spine surgery, posterior cervical spine surgery, multidisciplinary team (MDT)

## Abstract

Drug fever is a febrile reaction that emerges temporarily with the administration of a drug or a variety of drugs and disappears after cessation of the targeting agent. There are a few previous reports about drug fever, but they pertain mainly to patients accompanied by no surgical intervention. Based on the literature reviewed, drug fever in patients after posterior cervical spine surgery has never been mentioned before; therefore, we present a 56-year-old man diagnosed with drug fever after posterior cervical spine surgery for traumatic cervical myelopathy. Fortunately, his body temperature rapidly came down in 2 days after discontinuing the antibiotics. He was discharged after two more days of observation, and the patient recovered well without any further complaints. Early diagnosis of drug fever may greatly reduce inappropriate and potentially detrimental diagnostic and therapeutic interventions. For patients with persistent fever, if it happened days after surgery, particularly when it is without any infectious evidence, then it is necessarily important to consider a possible reason of drug-induced fever.

## Introduction

Drug-induced fever is defined as a febrile symptom that begins soon after the initiation of a drug and diminishes after ceasing the drug and, meanwhile, by exclusion of any other causes (1). Drugs that have been suspected or implicated in causing fevers include antimicrobials (2), which are the most common ones, anticonvulsants, antineoplastic, cardiovascular disease agents (3), nonsteroidal anti-inflammatory drugs (NSAIDs) (4), and less likely heparin (1) and favipiravir (5). However, most of the reported cases were not accompanied with surgical intervention and were mainly related to internal medical diseases. In our case, we demonstrated drug fever in a patient after posterior cervical spine surgery. Concomitantly, we reviewed reported cases within the past 10 years involving clinical characteristics and therapeutic outcomes to further demonstrate and better understand the features of drug-induced fever.

## Case presentation

**Figure 1:**
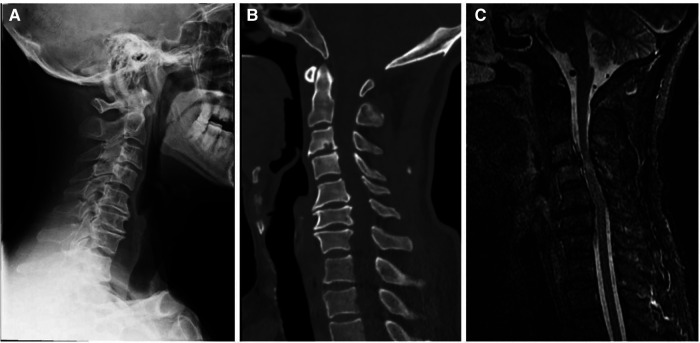
Preoperative x-ray (**A**), CT (**B**), and MRI (**C**) showed a diagnosis of traumatic cervical myelopathy.

**Figure 2:**
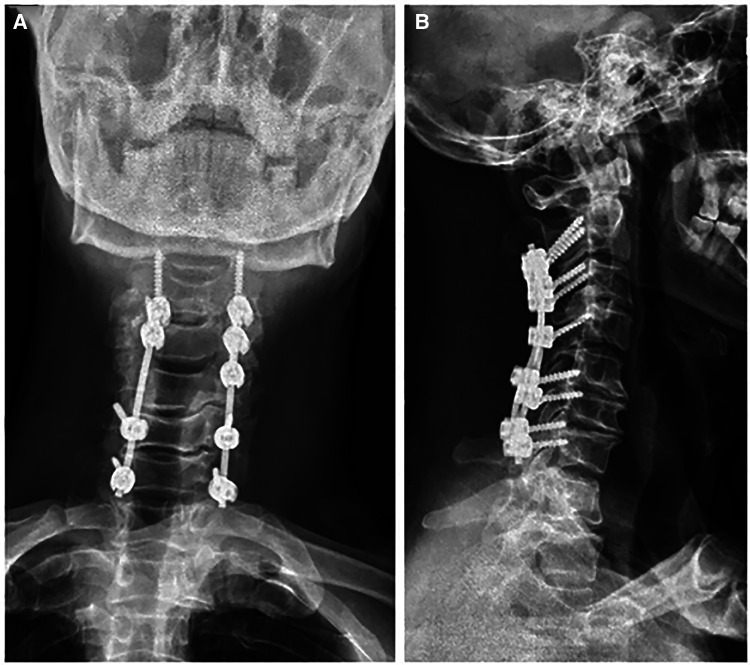
Postoperative x-ray [(**A**) anterio-posterially and (**B**) laterally] showed a satisfactory posterior fixation of cervical spine from C2–7.

**Figure 3:**
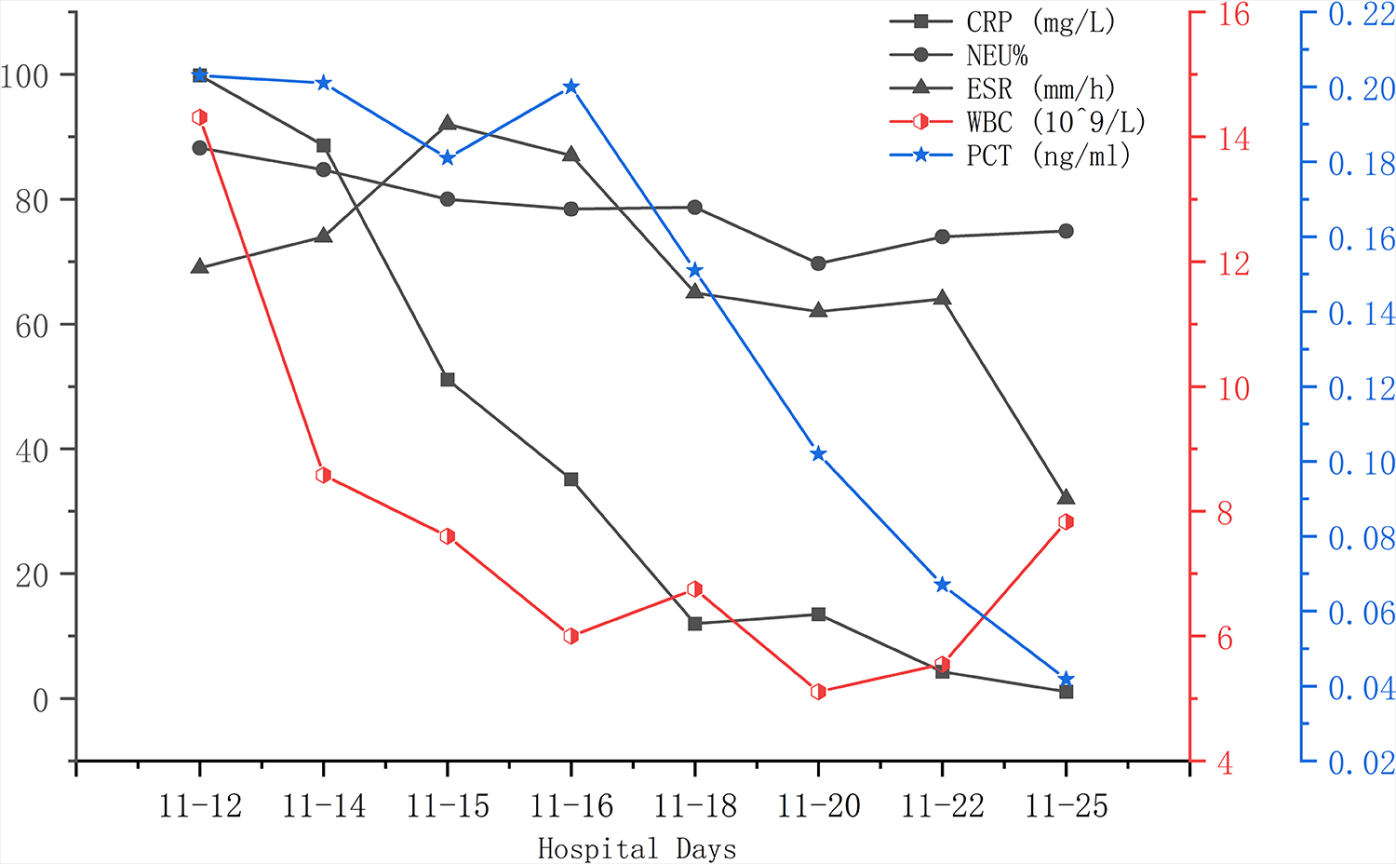
During this fever period (12 November was on postoperative day 8), elevated CRP, NEU%, ESR, and WBC gradually decreased to normal ranges. PCT was always within normal range.

**Figure 4:**
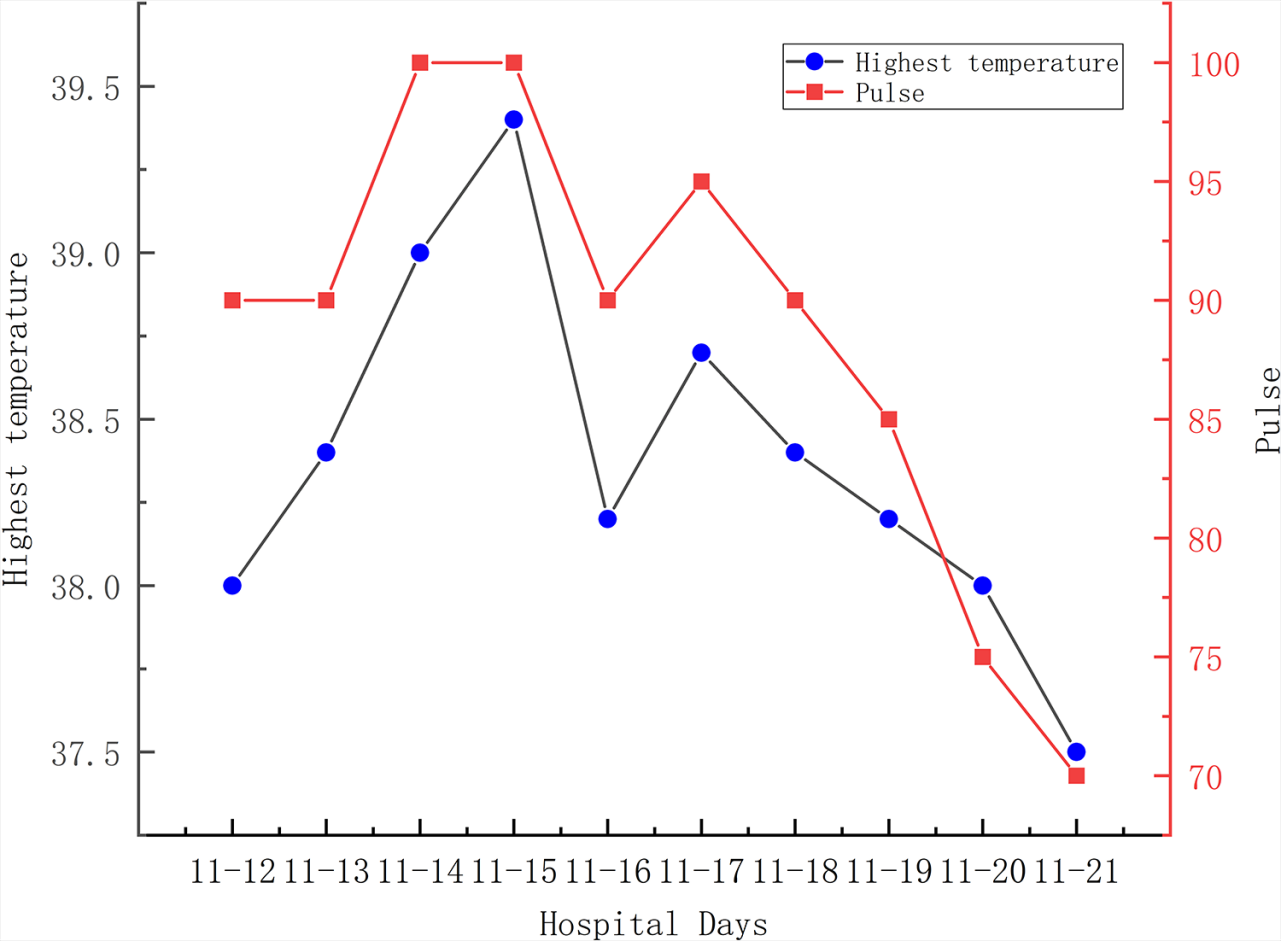
A relative bradycardia could be calculated as demonstrated in our case.

A 56-year-old man diagnosed with traumatic cervical myelopathy was admitted to our department (4 November) (Figure 1). His body weight was 62 kg, height was 168 cm, and BMI was 21.97 kg/m^2^. Dual x-ray bone absorptiometry was T < −2.0. Our patient did not have any medical or surgical history or any known drug allergies. Physical examination showed decreased muscle strength of the upper extremities; muscle strength of the lower extremities was normal; no incontinence of bladder or bowl function was detected. The Hoffmann sign was positive on both sides. Slight “panda's eyes” could be noticed. Moreover, multiple small abrasions were observed on his head, face, and limbs. After full preparation, he received posterior cervical decompression surgery. A general antibiotic of cefuroxime 1.5 g, b.i.d., was intravenously given. On postoperative day 2, white blood cell (WBC) was 7.31×10^9^/L (4–10), erythrocyte sedimentation rate (ESR) was 10 mm/h (0–20), C-reactive protein (CRP) was 7.1 mg/L (5–10), and procalcitonin (PCT) was 0.210 ng/ml (<0.5), which were all in normal range. He recovered smoothly; therefore, all other supplemental medicines were gradually stopped on postoperative day 3 except the antibiotic in case of skull base fracture and intracranial infection (although no obvious basilar fracture was detected on preoperative head CT) (Figure 2). However, his temperature rose abruptly to 38°C on the morning of postoperative day 8 (12 November) (Figure 4); then, for fear of infectious reasons, full infectious workup tests were performed, and the results showed the following: WBC: 14.31×10^9^/L (4–10), which decreased to normal range 2 days later; neutrophil% (NEU): 88.2 (50–70), which abated to normal range 10 days later; PCT: 0.203 ng/ml (<0.5), which was always in the normal range; ERS: 69 mm/h (0–20); and CRP: 99.8 mg/L (5–10), which decreased back to the normal 10 days later (Figure 3). Regular checking and dressing changes of the incision were performed during his recovery; no redness, swelling, secretion, or rebound pain were observed, but diagnosis of incisional infection still needs to be considered. Later that afternoon, we changed our antibiotic to piperacillin and tazobactam 2.25 g (8:1), q8h (12 November), intravenously injected. Nevertheless, the next morning, the patient's temperature rose again, and a widespread urticaria-like rash on the chest was observed. After giving an antifever medicine of paracetamol, his temperature decreased to normal, but it rose again that afternoon. Notably, his rash disappeared as his temperature decreased and vice versa. Our patient complained no other significant discomfort except intermittent fever. This kind of intermittent fever continued for three more days (15 November); during this period, what we were still most concerned about was the presence of infection. We later arranged chest CT, urine, and feces tests to exclude any potential infectious factors, but the results were all negative. Blood cultures were also taken for consecutively 3 days at the time his temperature raised up to 38.5°C; results later were all negative, and as his incision healed, we removed his stitches on postoperative day 11 (15 November). Under these circumstances, we organized a multidisciplinary team for him on the 9th day of fever and intermittent rash (21 November). Experts from the departments of respiratory diseases, urology, neurosurgery, and infectious diseases concluded that the available laboratory and imaging examinations combined with the patient's current symptoms and signs did not support infectious lesions. The department of pharmacology made the conclusion that the patient's surgical incision recovered smoothly and the stitches have been removed; further, no significant positive result was showed to support any infectious factors, but the patient continued to have intermittent fever for 9 days starting on 1 week of antibiotic treatment; furthermore, the Naranjo ADR probability scale and WHO-UMC scale of our patient were 7 showing *probable*/*likely*, respectively. It is therefore speculated that the possibility of drug fever induced by antibiotics of β-lactams, that is, cefuroxime and piperacillin and tazobactam should be considered, and antibiotics were recommended to be stopped. We then stopped the drugs (21 November). Fortunately, that afternoon, the highest temperature of our patient was 37.5°C (Figure 4); symptom of fever gradually decreased to normal status after 48 h of discontinuing of the drugs (23 November). He never developed another febrile episode during two more days of observation (25 November), He was later discharged in a stable condition and has recovered well without any further complaints.

## Discussion

Fever induced by drugs is a disorder characterized by a febrile symptom that is ephemerally ignited with the administration of a drug or a variety of drugs and later disappears after stopping the offending drugs. The diagnosis of drug fever is a deemed process of exclusion. Although this kind of fever resolves after extensive testing and, ultimately, cessation of the offending drugs, it is clinically difficult to ascertain the final accurate diagnosis, especially in postoperative, critical care, or burn patients (6). The reasons for what induced a drug fever are numerous and, in many cases, are not thoroughly investigated and understood. Many authorities have generally classified the reasons into six categories (1) Drug administration-related fever—a febrile reaction can be directly ignited by the process of administering a drug. (2) Fevers can sometimes be induced by contamination or due to the intrinsic effect of themselves. (3) Hypersensitivity reactions, a humoral response, were reported to be the most probable mechanism in mediating the process. (4) Altered thermoregulatory mechanisms—It is reported that altered thermoregulatory mechanisms could be disrupted by variety of drugs, either by increasing heat production or limiting heat dissipation. (5) Sometimes the pharmacologic effect of a drug itself is the cause of drug fever. For example, it is reported that the Jarisch–Herxheimer reaction is a classic example observed during antibiotic therapy for spirochetal diseases such as syphilis, leptospirosis, and borreliosis. (6) Idiosyncratic reactions—In certain patients, hereditable genetic predisposition could be the target of a febrile idiosyncratic drug (7–9).

The onset timing and pattern of the fever are mostly not useful in making an accurate diagnosis, but they really play an important auxiliary role in making a differentiating diagnosis. Accordingly, the average time it takes to onset is generally 7–10 days (10–12) but can also vary from hours to even several months (13), which is mostly consistent with our reviewed literature, ranging from 4 h (14) to 17 days (2, 15) (Table 1). After discontinuation of the offending drugs, the full resolution of fever generally takes 2–3 days, although it may still persist for days or even weeks if other manifestations of hypersensitivity come along with the fever, such as a widespread rash, or if the elimination rate of a certain agent remains low (16). Similarly, as is shown in our case and reviewed cases, they ranged from 2 days (1, 17) to 9 days (18) (Table 1).

**Table 1:** Reported cases discussing about drug-induced fever within current 10 years.

Authors	Year	Patients	Primary diseases	Relevant drugs	Onset of fever	Main symptoms and signs	Cessation time to normal temperature (days)
Linares et al. (17)	2011	65 years/male	Achilles tendon infection	Piperacillin/tazobactam	13 days later	Onset T: 39°C, shivering with rash and pruritus	2
Ochi et al. (18)	2011	38 years/male	MRSA infection	Teicoplanin	9 days later	T: 38–39°C	9
Zheng et al. (10)	2015	36 years/female	Spinal scoliosis	Piperacillin/tazobactam	3 days later	Onset T: 39°C	4
Shao et al. (11)	2015	62 years/male	Pneumonia	Tigecycline	7 days later	Onset T: 39°C with rash	3
Yuan et al. (12)	2016	48 years/female	Brucellosis	Doxycycline	7 days later	Onset T: 38°C	3
Yang et al. (15)	2017	40 years/female	Schizophrenia	Olanzapine	17 days later	Onset T: 39°C	5
Laun et al. (1)	2019	23 years/female	Burn injuries	Heparin	14 days later	T: 38.7–40.6°C	2–3
Yang et al. (15)	2019	66 years/male	Esophageal carcinoma	Imipenem/cilastatin	4 h later	Onset T: 38°C with shivering	Not specified
Kurita et al. (5)	2020	82 years/male	Covid-19	Favipiravir	7 days later	Onset T: 38°C	4
Xiao et al. (2)	2021	32 years/male	Pyrexia	Celecoxib	17 days later	Onset T: 38.2°C	2

Main symptoms and signs of drug-induced fever are as follows: fever usually starts at 38–39°C; in our patient, we noticed that the temperature rose within 15 min–1 h as the antibiotic was infused; relative was bradycardia lower than an expected number; and widespread cutaneous findings were mostly observed on the chest and back but also could be noticed on the face and extremities. There are many patterns of fever demonstrating themselves as follows: (1) Continuous fevers, in which temperature persists in a range from 39 to 40°C for days or weeks; remittent fevers, in which temperatures fluctuate within 2°C but are consistently higher than normal; intermittent fevers, in which high temperatures are randomly interrupted and replaced by normal temperatures during a day; and last but not the least, hectic fevers, also known as consuming fevers, which manifest as an M combination of intermittent and remittent fever. It is necessary to notice that hectic fever is the most common pattern observed in patients with drug fever, particularly because the use of antifever drugs and physic cooling therapy can easily change a primarily solid pattern (19). Relative bradycardia can sometimes be used as an indicative factor in predicting drug-induced fever. In order to get an approximate expected pulse response from a given temperature, we could use the last digit of the Fahrenheit temperature number (generally ≥100, 37.78°C) and decrease it by 1, later multiply this number by 10, and then add it to 100. Take a temperature of 100°F as an example; the appropriate expected pulse response would be calculated to approximately 90 beats/min. Any value less than this would be considered a relative bradycardia and therefore might play an important role in indicating a drug fever (9). However, it has been reported that relative bradycardia occurred in only 11% of patients (8). In our case, it is shown that the phenomenon of relative bradycardia could be calculated from the results because the actual pulse was mostly slower than an expected pulse rate (Figure 4). Cutaneous findings such as a widespread rash can also be observed in less than 30% of patients (16). In our case, fever, relative bradycardia, and cutaneous findings of a rash were all complained. Similarly, most of our reviewed cases showed the same symptoms and signs (Table 1).

Laboratory findings are often not very helpful in accurately determining a cause or understanding a relationship between a drug and the fever reaction. In patients with drug fever, the white blood cell count and ESR may be elevated along with eosinophilia, but these findings occur in less than 20% of cases, in which a similar result was shown in our case; however, in our case, the eosinophilia count and eosinophilia ratio were in the normal range (20, 21) (Figure 3).

Reported diagnostic criteria mainly are: (1) an onset central body temperature >37.5–38.0°C; (2) intravenous antibiotics for >3 days; (3) exclusion of infectious or other noninfectious causes of fever; and (4) rechallenge effect or defervescence after discontinuation of antibiotics (6, 9, 22). Our case conformed to most of the reported criteria, except that we did not perform a rechallenge test on our patients.

The only way to confirm if a patient has a drug-induced fever is to stop the potential offending drugs. The treatment initially engaged is often to discontinue the most potential drug first, followed sequentially by stopping other potentially targeted drugs if the fever continues (14). Nonetheless, in some situations, when the continued use of the drug is required and cannot be halted, switching to a chemically unrelated but functionally similar substitute may be selected if possible. There are more excruciating predicaments that some drugs do not have a satisfactory substitute, such as drugs in treating a particular type of cancer or under the circumstance of highly drug-resistant microbes. It was reported to be acceptable to pretreat these patients with anti-anaphylactic drugs such as antihistamines, corticosteroids, and/or prostaglandin inhibitors while still remaining vigilant for further reactions of hypersensitivity (16). If a patient receives a therapy with multiple agents, and furthermore, these agents are all possibly the causes of fever, then theoretically, the cessation of all nonessential drugs should be initiated first. If necessary, drugs may be reintroduced cautiously later, after the fever has completely subsided, with the most highly suspected drugs excluded (19). It is shown that antibiotics, particularly categories of β-lactams, are increasingly associated with a higher incidence of drug fever. Similarly, in our case, the patient suffered from drug fever induced by cefuroxime and piperacillin/tazobactam, so we have recommended to our patient that it is demanding to avoid using as many categories of these medicines as possible.

The reporting of this study conforms to the CARE guideline (23).

## Conclusion

For patients with persistent fever, especially if it happened days after spine or any other surgery, it is fundamentally important to perform an infectious workup as well as an investigation to differentiate other potential etiologies. However, if there are no overt abnormalities and the symptom of fever continues despite the negative results of the laboratory findings, a multidisciplinary team (MDT) would be highly recommended. It is important and necessary to consider a possible reason of drug-induced fever.

## Data Availability

The original contributions presented in the study are included in the article/Supplementary Material. Further inquiries can be directed to the corresponding author.

## References

[1] Laun J Laun K Farooqi A Smith DJ. Heparin-induced fever: a case report and literature review. J Burn Care Res. (2019) 40(5):723–4. 10.1093/jbcr/irz064 30977800

[2] Xiao J Jia SJ Wu CF. Celecoxib-induced drug fever: a rare case report and literature review. J Clin Pharm Ther. (2022) 47(3):402–6. 10.1111/jcpt.13490 34287995

[3] Dominguez EA Hamill RJ. Drug-induced fever due to diltiazem. Arch Intern Med. (1991) 151:1869–70. 10.1001/archinte.1991.00400090141026 1888256

[4] Mandell B Shen HS Hepburn B. Letter: fever from ibuprofen in a patient with lupus erythematosus. Ann Intern Med. (1976) 85:209–10. 10.7326/0003-4819-85-2-209 942146

[5] Kurita T Ishida K Muranaka E Sasazawa H Hase R. A favipiravir-induced fever in a patient with COVID-19. Intern Med. (2020) 59:2951–3. 10.2169/internalmedicine.5394-20 33191372 PMC7725626

[6] Tishler M, Yaron M. Drug Fever due to sulfasalazine in rheumatoid arthritis-a report of two cases. *J Clin Rheumatol*. (1997) 1:64. 10.1097/00124743-199702000-00016 19078124

[7] Roush MK Nelson KM. Understanding drug-induced febrile reactions: when a fever of unknown etiology develops it is important to consider drug fever to avoid delays additional costs of treatment. Am Pharm. (1993) 33(10):39–42. 10.1016/S0160-3450(15)30635-8 8237783

[8] Mackowiak P. Drug fever: a critical appraisal of conventional concepts. An analysis of 51 episodes in two Dallas hospitals and 97 episodes reported in the English literature. Ann Intern Med. (1987) 106:728–33. 10.7326/0003-4819-106-5-728 3565971

[9] Johnson DH Cunha BA. Drug fever. Infect Dis Clin North Am. (1996) 10:85–91. 10.1016/S0891-5520(05)70287-7 8698996

[10] Zheng L Shen J Li Q Chan M Wu W. Drug fever induced by piperacillin/tazobactam in a scoliosis patient: a case report. Medicine. (2015) 94:e1875. 10.1097/MD.0000000000001875 26579799 PMC4652808

[11] Shao QQ Qin L Ruan GR Chen RX Luan ZJ Ma XJ. Tigecycline-induced drug fever and leukemoid reaction: a case report. Medicine. (2015) 94:e1869. 10.1097/MD.0000000000001869 26559254 PMC4912248

[12] Yuan HL Lu NW Xie H Zheng YY Wang QH. Doxycycline-induced drug fever: a case report. *Infect Dis (Lond)*. (2016) 48:844–6. 10.1080/23744235.2016.1195915 27339030

[13] Mackowiak PA. Drug fever: mechanisms, maxims and misconceptions. Am J Med Sci. (1987) 294:275–86. 10.1097/00000441-198710000-00011 3310641

[14] Yang J, Wang Q, Wang S, Zhang Y, Wang Z. Unusual drug fever caused by imipenem/cilastatin and a review of literature. *Heart Surg Forum*. (2019) 22:E119-23. 10.1532/hsf.2141 31013221

[15] Yang CH Chen YY. A case of olanzapine-induced fever. Psychopharmacol Bull. (2017) 47:45–7. PMID: 28138204 10.64719/pb.4356 PMC5274531

[16] Patel RA Gallagher JC. Drug fever. Pharmacotherapy. (2010) 30:57–69. 10.1592/phco.30.1.57 20030474

[17] Linares T Fernández A Soto MT Escudero E Gacías L. Drug fever caused by piperacillin-tazobactam. J Investig Allergol Clin Immunol. (2011) 21:250. PMID: 21548459

[18] Ochi H Wada K Okada H Kohara N Nakatani T. The persistence of drug-induced fever by teicoplanin—a case report. Int J Clin Pharmacol Ther. (2011) 49:339. 10.5414/CP201430 21543038

[19] Patricia A Tabor. Drug-induced fever. Drug Intell Clin Pharm. (1986) 20:413–20. 10.1177/106002808602000601 3522163

[20] Lipsky BA Hirschmann JV. Drug fever. JAMA. (1981) 245:851–4. 10.1001/jama.1981.03310330041024 7463680

[21] Vodovar D Beller CL Mégarbane B Louet LL. Drug fever. Adverse Drug React Bull. (2014) 284:1–4. 10.1097/FAD.0000000000000002

[22] Kirsten L Karina JJ Carsten P Andrej T Renz N. Antibiotic-induced fever in orthopaedic patients—a diagnostic challenge. Int Orthop. (2018) 42(8):1775–81. 10.1007/s00264-018-3909-8 29600426

[23] Gagnier JJ Kienle G Altman DG Moher D Sox H Riley D, CARE Group. The CARE guidelines: consensus-based clinical case reporting guideline development. Headache. (2013) 10:1541–7. 10.1111/head.12246 24228906 PMC3844611

